# A Novel Toolkit of SARS-CoV-2 Sub-Genomic Replicons for Efficient Antiviral Screening

**DOI:** 10.3390/v17050597

**Published:** 2025-04-23

**Authors:** Maximilian Erdmann, Peter A. C. Wing, Isobel Webb, Maia Kavanagh Williamson, Tuksin Jearanaiwitayakul, Edward Sullivan, James Bazire, Iart Luca Shytaj, Jane A. McKeating, David A. Matthews, Andrew D. Davidson

**Affiliations:** 1 School of Cellular and Molecular Medicine, University of Bristol, Bristol BS8 1TD, UK; max.erdmann.2017@bristol.ac.uk (M.E.); isobel.webb@bristol.ac.uk (I.W.); tuksin.jea@nmu.ac.th (T.J.); edward.sullivan@bristol.ac.uk (E.S.); james.bazire@crick.ac.uk (J.B.); luca.shytaj@bristol.ac.uk (I.L.S.); d.a.matthews@bristol.ac.uk (D.A.M.); 2 Chinese Academy of Medical Sciences Oxford Institute, University of Oxford, Oxford OX3 7BN, UK; peter.wing@ndm.ox.ac.uk (P.A.C.W.); jane.mckeating@ndm.ox.ac.uk (J.A.M.); 3 Nuffield Department of Medicine, University of Oxford, Oxford OX3 7BN, UK; 4 Faculty of Science, Mahidol University, Bangkok 10400, Thailand

**Keywords:** SARS-CoV-2, RNA replicon, antiviral, SARS-CoV-2 nsp1

## Abstract

SARS-CoV-2 is classified as a containment level 3 (CL3) pathogen, limiting research access and antiviral testing. To address this, we developed a non-infectious viral surrogate system using reverse genetics to generate sub-genomic replicons. These replicons contained the nsp1 mutations K164A and H165A and had the spike, membrane, ORF6, and ORF7a coding sequences replaced with various reporter and selectable marker genes. Replicons based on the ancestral Wuhan Hu-1 strain and the Delta variant of concern were replication-competent in multiple cell lines, as assessed by *Renilla* luciferase activity, fluorescence, immunofluorescence staining, and single-molecule fluorescent in situ hybridization. Antiviral assays using transient replicon expression showed that remdesivir effectively inhibited both replicon and viral replication. Ritonavir and cobicistat inhibited Delta variant replicons similarly to wild-type virus but did not inhibit Wuhan Hu-1 replicon replication. To further investigate the impact of nsp1 mutations, we generated a recombinant SARS-CoV-2 virus carrying the K164A and H165A mutations. The virus exhibited attenuated replication across a range of mammalian cell lines, was restricted by the type I interferon response, and showed reduced cytopathic effects. These findings highlight the utility of sub-genomic replicons as reliable CL2-compatible surrogates for studying SARS-CoV-2 replication and drug activity mechanisms.

## 1. Introduction

Severe acute respiratory syndrome coronavirus 2 (SARS-CoV-2) is the third highly pathogenic human coronavirus (CoV) to emerge in the last two decades [1,2,3,4]. Like SARS-CoV and Middle Eastern respiratory syndrome (MERS)-CoV, it is an enveloped RNA virus belonging to the *Betacoronavirus* genus of the *Coronaviridae* family [1]. SARS-CoV-2 is the causative agent of ‘coronavirus disease 19’ (COVID-19) [5] first detected in Wuhan, China, in December 2019. SARS-CoV-2 rapidly spread, resulting in the declaration of the ongoing COVID-19 pandemic in March 2020 [5,6]. Like all coronaviruses, SARS-CoV-2 has a capped, positive-sense RNA genome of approximately 30 kb [7]. At the 5′-end of the genome, there is a highly structured untranslated region (UTR) followed by open reading frames (ORF)1a and ORF1ab, that encode the large polyproteins 1a and 1ab, respectively [2,8]. The two polyproteins are processed by viral encoded proteases to yield 16 non-structural proteins (nsp1-16) which are highly conserved across coronaviruses [9]. The nsps form the replication-transcription complex that transcribes a set of nested sub-genomic RNAs (sgRNA) from transcriptional regulatory sequences (TRS) preceding the smaller 3′-terminal ORFs during genome replication via discontinuous transcription [10]. The sgRNAs encode structural proteins (spike (S), membrane (M), envelope (E) and nucleocapsid (N)) and small accessory proteins that modulate the host immune response [7,11,12].

Reverse genetics enables the modification of viral genomes to investigate how specific genotypes influence viral phenotypes [13]. Several reverse genetic systems have been established for CoVs [14,15]. In this study, a yeast-based system [16] was used to establish cDNA clones corresponding to the SARS-CoV-2 genome or sub-genomes. RNA transcripts generated from full-length cDNA clones in vitro can be transfected into permissive host cells. In the presence of the N protein, this process leads to the production of infectious virions. By contrast, transfection of in vitro RNA transcripts generated from sub-genomic cDNA clones, which lack essential viral structural genes, results in the production of replication competent RNA sub-genomes (termed replicons), which cannot assemble into infectious particles.

The development of CoV replicon systems dates back to the early 2000s, with the establishment of reverse genetics systems for various CoVs [17,18,19,20]. The first CoV replicon system was developed in 2004 for human CoV-229E (HCoV-229E) [21]. Sub-genomic CoV replicons, both DNA and RNA launched, have been developed using the minimal requirements for replication; the 5′/3′ UTRs and ORF1ab [14,21,22]. Expression of the N protein was also required, and achieved either in cis, by including the N-TRS and N coding sequence in the replicon, or in trans, using heterologous expression [23]. Such minimal replicons allow the study of the viral genes that remain, but to investigate other aspects of the virus life cycle, such as particle formation or the role of accessory proteins, the replicon needs to be expanded to include more of the viral genome [14].

As the introduction of replicon genomes into cells (via large DNA plasmids/in vitro transcripts) is technically demanding and relatively inefficient, the most practical systems replace an existing viral gene; most commonly S, with an antibiotic resistance gene to enable selection of stable replicon cell lines which can be used to analyse compounds or host–virus interactions [22]. This approach has been applied to generate stable replicon cell lines for HCoV-229E, SARS-CoV and MERS-CoV [14,21,24], but also for other species such as dengue virus [25]. For viruses like SARS-CoV-2, where work is restricted to containment level (CL)-3 and -4 laboratories, sub-genomic viral replicons have proven to be valuable tools [22,24,26,27]. Current SARS-CoV-2 replicon systems mainly rely on transfection of in vitro RNA transcripts (derived from replicon cDNA clones under the control of a T7 RNA polymerase promoter) into permissive cells. This approach prevents background expression of reporter genes [28,29,30,31,32], and provides a convenient amplification step.

In many studies a gene encoding a reporter-selectable marker fusion protein is driven from the S-TRS [29,31], or a reporter from the S-TRS and a resistance gene from a different ORF (e.g., E-TRS) [28]. Some studies have opted for an approach retaining most viral genes [28,32]. However, a common challenge across studies is poor transfection efficiency of most cell lines tested with replicon RNA transcripts and the failure to establish stable replicon-containing cell lines [28,30]. Whether designed as minimal replicons containing only ORF1ab and the sequences downstream of the N-TRS [29,31], or lacking only structural proteins [28], several studies have suggested that the replicon itself exerts high cytotoxicity [28,29]. This is not surprising, given that several of the accessory genes (mainly ORF3a, ORF6, ORF7 and ORF8) and certain non-structural genes are potent immunomodulators and induce apoptosis [33,34,35,36,37]. Among the main functions of ORF6 are the prevention of mRNA export from the nucleus, and ORF7a was found to inhibit the type I interferon (IFN) response and promote apoptosis [34,36,37].

Nonstructural protein 1 (nsp1) is the first SARS-CoV-2 protein generated by processing of the ORF1a polyprotein and is known to inhibit host translation [38,39,40]. Although it has low sequence conservation among betacoronaviruses (it is absent in gamma- and deltacoronaviruses), its ability to interfere with host innate immunity at the transcriptional and/or translational level is highly conserved across CoVs (e.g., for murine hepatitis virus (MHV), SARS-CoV and MERS-CoV) [39,40,41,42]. The nsp1 amino acids K164 and H165 are essential for inhibition of host cell translation [38,43,44,45]. Studies have shown that the double substitution K164A/H165A results in the complete loss of SARS-CoV and SARS-CoV-2 nsp1 function with respect to translation shut-off [38,43,44]. Introduction of the double substitution into SARS-CoV-2 replicons reduced their cytotoxicity [32], and in one case, led to the recovery of cells stably harbouring a replicon [46]. In this study, the K164A/H165A substitutions were incorporated into the nsp1 coding sequence to potentially attenuate replicon cytotoxicity. These mutations were also introduced into a recombinant infectious clone to assess their effect on viral infection.

In this study, we designed and tested a series of sub-genomic SARS-CoV-2 replicon cDNA clones, with the goal of developing an RNA launched replicon that could be stably maintained in cells. Unlike previous work, our approach included replicons derived not only from the ancestral B.1 isolate (Wuhan-Hu-1) but also from the Delta variant of concern (VOC). To reduce cytotoxicity associated with replicon expression, we engineered replicons carrying the nsp1 K164A/H165A substitutions. The constructs retained most viral genes, except for the S and M genes, which were replaced with puromycin resistance and *Renilla* luciferase genes, respectively. In addition, either ORF6 or ORF7a was replaced with the fluorescent reporter genes mNeonGreen or mScarlet. A Vero cell line overexpressing angiotensin-converting enzyme 2 (ACE2) and transmembrane serine protease 2 (TMPRSS2) was identified that supported high-efficiency transfection compared to other cell lines tested and was permissive for transient replication of in vitro transcribed RNA replicons. However, despite these improvements, stable replicon cell lines could not be established by puromycin selection. The replicons developed here, which express both enzymatic and fluorescent reporters, provide sensitive and versatile surrogate readouts for monitoring SARS-CoV-2 RNA replication. They are well suited for use in reporter assays to distinguish the effects of antiviral compounds on viral entry and egress versus intracellular replication.

## 2. Materials and Methods

### 2.1. Bacteria and Yeast

OneShot^®^ Top10 Electrocomp™ *Escherichia coli* (Invitrogen™, Thermo Fisher Scientific, Waltham, MA, USA) were used to propagate the GeneArt™ pYES1L vector (Invitrogen™, Thermo Fisher Scientific) containing SARS-CoV-2 replicon cDNA clones and grown overnight at 37 °C either on lysogeny broth (LB)-agar plates or shaking in LB media supplemented with spectinomycin at 50 µg/mL and 100 µg/mL, respectively. MaV203 competent yeast cells (MATα; leu2-3,112; trp1-901; his3Δ200; ade2-101; cyh2R; can1R; gal4Δ; gal80Δ; GAL1::lacZ; HIS3UASGAL1::HIS3@LYS2; SPAL10UASGAL1::URA3; Invitrogen™, Thermo Fisher Scientific) were used for yeast-based transformation-associated recombination (TAR) and propagation of inserts contained in the pYES1L vector. Yeast was grown on 2% (*w*/*v*) agar plates containing yeast nitrogen base without amino acids (6.8 g/L, Sigma-Aldrich, Merck, Darmstadt, Germany) supplemented with yeast synthetic drop-out medium minus tryptophan (1.92 g/L, Sigma Aldrich) and 2% (*w*/*v*) D-(+)-glucose (YSM-Trp plates) at 30 °C.

### 2.2. Generation of SARS-CoV-2 cDNA Fragments for TAR

A number of approaches were used to produce SARS-CoV-2 cDNA fragments for the assembly of replicon and full-length SARS-CoV-2 cDNA clones by TAR, as previously described [16], as follows.

**Figure 1:**
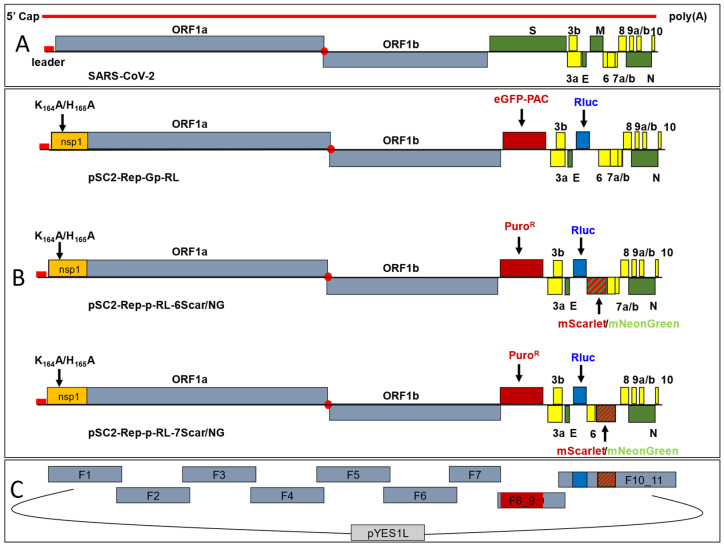
Construction of SARS-CoV-2 replicon clones. (**A**) Schematic of the SARS-CoV-2 genome and ORFs. (**B**) Replicon cDNA BAC/YAC constructs. For pSC2-Rep-Gp-RL, the S and M genes were replaced with sequences encoding an eGFP-pac fusion protein and RLuc, respectively. Mutations encoding amino acid changes K164A/H165A were introduced into the nsp1 sequence. pSC2-Rep-Gp-RL was modified further by replacing the eGFP-pac sequence with the *pac* gene coding sequence alone (Puro^R^) and ORF6 and ORF7a with either the mNeonGreen or mScarlet coding sequences, resulting in the pSC2-Rep-p-RL-6Scar/NG and pSC2-Rep-p-RL-7Scar/NG replicon clones, respectively. (**C**) TAR assembly of replicon cDNA genomes. Overlapping cDNA fragments spanning the genome with 70 bp terminal end-homology were assembled into BAC/YAC shuttle vector pYES1L. Fragments F8_9 and F10_11 contain replicon-specific gene replacements.

(a) Synthetic DNA: Initially, nine cDNA fragments with 70 bp end-terminal overlaps were used to assemble a SARS-CoV-2 replicon clone based on the Wuhan-Hu-1 genome sequence (GenBank accession: NC_045512, Table S1). The cDNA fragments were produced by GeneArt™ synthesis (Invitrogen™, Thermo Fisher Scientific) as cDNA inserts in sequence-verified, stable plasmid clones. The 5′ terminal cDNA fragment was modified to contain 70 nucleotides corresponding to nucleotides (nts) 9311–9380 of the pYES1L vector, a T7 RNA polymerase promoter and an extra “G” nucleotide immediately upstream of the SARS-CoV-2 5′-terminal genome sequence, whilst the 3′-terminal cDNA fragment was modified such that the 3′ end of the SARS-CoV-2 genome was followed by a stretch of 33 “A”s followed by the unique restriction enzyme site *Asc*I and nts 1–70 of the pYES1L vector. The first seven cDNA fragments (from the 5’end) spanned nts 1–20,090 of the SARS-CoV-2 genome. The two remaining cDNA fragments spanned nts 20,021–29,903 of the SARS-CoV-2 sequence with the following exceptions: nts 21,653–25,384, encoding the S protein, were replaced with a 1359 nt sequence encoding an enhanced green fluorescence protein (eGFP)-puromycin N-acetyl transferase (pac) fusion protein, and nts 26,523–27,191 encoding the M protein, were replaced with a 936 nt sequence encoding *Renilla* luciferase (RLuc). The cDNA fragment contained in each clone was PCR amplified using gene specific primer pairs and the Platinum SuperFi II mastermix (Invitrogen™, Thermo Fisher Scientific) following the manufacturer’s instructions. The location of the fragments and primers and sequences of introduced genes are shown in Table S2.
(b) Overlap PCR mutagenesis: Modification of the synthetic replicon cDNA clones to introduce site-specific mutations, gene substitutions and a hepatitis delta virus ribozyme sequence followed by a T7 RNA polymerase terminator sequence (see Table S2, a kind gift from Professor Arvind Patel, MRC-University of Glasgow Centre for Virus Research) immediately downstream of the 3’end poly-A tail was performed by overlap-PCR (OL-PCR) mutagenesis. Template DNA fragments for OL-PCR were first produced as overlapping sub-fragments (20–30 nt overlaps) using an outer primer and an internal mutagenesis primer (primers shown in Table S3). The first-round PCR sub-fragments were purified by extraction from an agarose gel using a GeneJET Gel Extraction Kit (Thermo Scientific™, Thermo Fisher Scientific). A total mass of 10–15 ng of sub-fragments at a 1:1 molar ratio was then used as a template for OL-PCR using the forward and reverse outer primers. Assembly of more than two PCR fragments was performed stepwise.
(c) Viral RNA extraction and reverse-transcriptase (RT)-PCR: For production of a SARS-CoV-2 Delta VOC replicon, fragments 2, 4, 5 and 6, corresponding to the Wuhan-Hu-1 virus genome (Figure 1), were swapped for the corresponding cDNA fragments generated by RT-PCR from Delta VOC extracted RNA. For production of a chimeric SARS-CoV-2 containing the S gene from the Delta VOC in the Wuhan-Hu-1 virus genome, fragments 8 and 9 were generated by RT-PCR from Delta VOC extracted RNA. Viral RNA was extracted from 140 µL of virus stock (SARS-CoV-2 Delta VOC, GISAID ID: EPI_ISL_15250227) using a QIAamp Viral RNA Mini Kit (Qiagen, Hilden, Germany) following the manufacturer’s instructions. RT-PCR was performed using 1 µL of eluted RNA and a SuperScript™ IV One-Step RT-PCR System (Invitrogen™, Thermo Fisher Scientific) as described by the manufacturer.
(d) HiFi DNA assembly: To produce SARS-CoV-2 Delta VOC replicon clones, the viral sequence from nts 20,021–29,903 was replaced with either of two complementary DNA (cDNA) fragments in which the S and M gene coding sequences were replaced with those of the *pac* and *Rluc* genes, and either the ORF6 or ORF7a coding sequences were replaced with those of the mScarlet and mNeonGreen genes, respectively. Assembly of the two cDNA fragments was performed using five overlapping cDNA fragments containing the VOC lineage-defining mutations and replicon-specific gene replacements (see Tables S4 and S5) using a NEBuilder^®^ HiFi DNA Assembly Master Mix (NEB, Ipswich, MA, USA) according to the manufacturer’s recommendations. They were assembled in the vector pYES1L, the assembly reactions purified and electroporated into One Shot™ TOP10 Electrocomp™ *E. coli* (Invitrogen™, Thermo Fisher Scientific). Two colonies were picked, screened for correct assembly and used as PCR templates. The resulting fragments were used for TAR assembly.

All cDNA fragments generated by PCR/RT-PCR were purified using a GeneJET PCR Purification kit (Thermo Fisher Scientific) following the manufacturer’s instructions prior to use in TAR. The concentration of purified DNA fragments was determined by spectrophotometry using a NanoDrop One (Thermo Scientific™, Thermo Fisher Scientific). The regions encompassed by the cDNA inserts and the sequences of the primer pairs and 5′ and 3′ cDNA fragment modifications are shown in Tables S1–S6.

### 2.3. TAR Assembly in Yeast

SARS-CoV-2 replicon cDNA fragments were assembled into full-length replicon cDNA clones by TAR assembly using the GeneArt™ High-Order Genetic Assembly System (Invitrogen™, Thermo Fisher Scientific) according to the manufacturer’s instructions. Briefly, 200 ng of each cDNA fragment was mixed with 100 ng of the pYES1L bacterial/yeast artificial chromosome (BAC/YAC) shuttle vector with Sapphire™ Technology (Invitrogen™, Thermo Fisher Scientific) and the volume reduced to 5–10 µL using a SpeedVac™ (Savant, Thermo Fisher Scientific); 100 µL of thawed MaV203 competent yeast was added to the tube, followed by 600 µL of polyethylene glycol/lithium acetate solution. The solutions were mixed gently and incubated at 30 °C for 30 min, followed by the addition of 35.5 µL of dimethyl sulfoxide and incubation at 42 °C for 20 min. The yeast was then plated on YSM-Trp plates and grown at 30 °C until colonies were visible.

### 2.4. Yeast Colony Screens

Correctly assembled replicon clones were identified by PCR screening of either yeast colonies directly or yeast cell lysates. For direct colony PCR, a yeast colony was picked from a YSM-Trp plate, resuspended in 100 µL of ultra-pure water and 1 µL used as a template for PCR. Yeast cell lysates were prepared following an adjusted GC preparation method [47] as follows. A yeast colony was picked from a YSM-Trp plate and resuspended in 100 µL of 5% *w*/*v* Chelex-100 (Sigma-Aldrich). Half the sample volume of acid-washed glass beads were added and the sample vortexed at maximum speed for 4 min at room temperature. The lysate was then heated at 99 °C for 2 min, followed by centrifugation at 13,000× *g* for 1 min; 50 µL of supernatant was transferred into a new tube and 1 µL used as template for PCR. Correct assembly of the YAC clones was verified by amplifying the overlapping junctions between the fragments by multiplex PCR, using two sets of multiplex primers and the Platinum SuperFi II PCR Master Mix (Invitrogen™, Thermo Fisher Scientific). The primers used in each multiplex reaction are shown in Table S7.

### 2.5. Transformation of E. coli with BAC/YAC Shuttle Plasmids

Correctly assembled SARS-CoV-2 replicon BAC/YAC clones were prepared for bacterial transformation as described in the GeneArt™ High-Order Genetic Assembly System protocol. An *E. coli* Pulser™ Transformation Apparatus (BioRad, Hercules, CA, USA) was used to electroporate One Shot™ TOP10 Electrocomp™ *E. coli* (Invitrogen™, Thermo Fisher Scientific) in a 0.2 cm cuvette. Electroporation was performed using conditions of 2.5 kV, 200 Ω and 25 µF. Immediately after electroporation, 500 µL of S.O.C. medium (Invitrogen™, Thermo Fisher Scientific) was added and the cells incubated at 37 °C for 1 h with shaking. Positive clones were selected by overnight growth on LB-spectinomycin (100 µg/mL) plates at 37 °C.

### 2.6. BAC/YAC Purification

Large-scale purification of BAC/YAC plasmid DNA was performed using a NucleoBond^®^ Xtra BAC (Macherey-Nagel, Düren, Germany) kit. Single *E. coli* colonies were picked, resuspended in 2 mL of LB and used to inoculate 500 mL of LB media containing spectinomycin (50 µg/mL). After overnight growth at 37 °C with shaking, bacteria were harvested by centrifugation at 6000× *g* for 30 min. Cell pellets were either frozen at −80 °C or immediately used for plasmid isolation according to the manufacturer’s protocol. The final DNA pellets were dissolved in ultra-pure water and the DNA yield and purity were determined by spectrophotometry.

### 2.7. Preparation of In Vitro RNA Transcripts

T7 polymerase promoter-driven in vitro RNA transcription from replicon/virus-encoding BAC/YAC clones and a construct containing a codon-optimised SARS-CoV-2 N gene sequence, under control of a T7 promoter (Table S5), were performed using a RiboMAX™ Large Scale RNA Production System-T7 (Promega, Madison, WI, USA). When required, the BAC/YAC/plasmid constructs were linearised prior to in vitro transcription using a unique *Asc*I cleavage site immediately downstream of the polyA sequences. The linearised DNA constructs were then purified by phenol/chloroform extraction followed by sodium acetate/ethanol precipitation. Constructs containing a 3′-terminal hepatitis delta virus ribozyme and T7 transcription terminator were transcribed directly from purified BAC/YAC DNA. For a 50 µL reaction, 1.5 µg of BAC/YAC/plasmid DNA template was incubated with 7.5 mM of ATP, CTP, UTP and 1.5 mM GTP. m7G(5′)G RNA Cap Analog (NEB) or Anti-Reverse Cap Analogue (NEB) was added to a final concentration of 3 mM followed by the addition of 5 µL of T7 Enzyme Mix and 50 U RNasin RNase Inhibitor (Promega). To produce transcripts for mock transfections, the GTP concentration was increased to 7.5 mM and cap analogues were not added. The reaction mixes were then incubated at 30 °C for 3 h. After incubation, the reaction mix was treated with RQ1 RNase-Free DNase to degrade template DNA. The reaction mix was then either used directly for electroporation or purified by lithium chloride precipitation, followed by three 75% ethanol washes, and resuspended in ultra-pure water. Both were stored at −80 °C.

### 2.8. Cell Lines

Human Caco-2 cells (ATCC^®^ HTB-37™; a kind gift from Professor Darryl Hill, University of Bristol) were obtained from the American Type Culture Collection and used to produce Caco-2 cells constitutively expressing SARS-CoV-2 N protein (Caco2-N). Human lung adenocarcinoma cells, Calu-3 (HTB-55 ™), were a kind gift from Prof Miles Carroll, University of Oxford. Human liver epithelial Huh7.5 cells [48] were a kind gift from Professor Mark Harris, University of Leeds. An African green monkey kidney cell line (Vero E6, ATCC CRL-1586™) modified to constitutively express human TMPRSS2 (VeroE6/TMPRSS2 (VTN)) [49] was obtained from NIBSC, UK. Vero E6 cells and human A549 (ATCC CRL-185™) cells modified to constitutively express ACE2 and TMPRSS2 (VeroE6/ACE2/TMPRSS2 (VAT) and A549/ACE2/TMPRSS2 (AAT)) cells, respectively, [50] were a kind gift from Dr Suzannah Rihn, MRC-University of Glasgow Centre for Virus Research. A baby hamster kidney 21 (BHK-21) cell line modified to constitutively express ACE2 and the SARS-CoV-2 N protein (BHK/ACE2/N (BAN)) [50] was a kind gift from Dr Arvind Patel, MRC-University of Glasgow Centre for Virus Research. A human bronchial epithelium cell line BEAS-2B (ATCC CRL-3588™) modified to express ACE2 (BEAS-2BA/ACE2 (BEAS-2BA)) was a kind gift from Prof Stuart Neil and Dr Harry Wilson, King’s College London.

All cells were maintained in Dulbecco’s Modified Eagle’s medium, containing 4.5 g/L D-glucose, and GlutaMAX™ (DMEM, Gibco™, Thermo Fisher Scientific) supplemented with 1 mM sodium pyruvate (Sigma-Aldrich) and 10% (*v*/*v*) foetal bovine serum (FBS, Gibco™, Thermo Fisher Scientific), except for Caco-2N and Calu-3 cells, which were maintained in Eagle’s Minimal Essential medium containing GlutaMAX™ (MEM, Gibco™, Thermo Fisher Scientific) supplemented with 1 mM sodium pyruvate (Sigma-Aldrich), 10% (*v*/*v*) FBS and 0.1 mM non-essential amino acids (Gibco™, Thermo Fisher Scientific). All cell lines were grown at 37 °C in a humidified incubator in 5% CO_2_. To establish air–liquid interface (ALI) cultures, Calu-3 cells were seeded onto the apical side of 6.5 mm PET Transwell^®^ inserts (0.4 μm pore size, Corning) at a density of 20,000 cells per insert in 300 μL of media. The basal compartment received 600 μL of media, and the cells were cultured submerged for seven days, allowing the monolayer to reach 100% confluence. At this point, the apical media was removed, and the inserts were maintained with basal media only for an additional seven days, with media changes every three days before infection.

### 2.9. Mammalian Cell Electroporation and Replicon Transfection

Electroporation was carried out using a Neon^®^ Transfection System (Invitrogen™, Thermo Fisher Scientific) following the manufacturer’s protocol for adherent cell lines (electroporation conditions for each cell line are shown in Table S8). Briefly, the cells to be transfected were grown to 60–80% confluence, detached using trypsin, harvested by centrifugation, and then washed in phosphate-buffered saline (PBS) before being resuspended in resuspension buffer at a density of 1 × 10^7^ cells/mL; 110 µL of cells were then mixed with 5–15 µg of replicon in vitro RNA transcripts and 2 µg of N gene in vitro RNA transcripts in a total volume of 11 µL. Alternatively, up to 9.9 µL of replicon in vitro RNA transcription reaction mixture was used instead of lithium chloride-purified RNA. Immediately after electroporation, cells were resuspended in complete warm growth media. Cells were then plated at an appropriate density after resting at 37 °C for 10 min.

### 2.10. Luciferase Assay

Assays to measure RLuc activity were performed using a *Renilla* Luciferase Assay System (Promega) following the manufacturer’s protocol. In short, cells were prepared and seeded in the appropriate culture vessels. To harvest the cells, the media was removed, and cells washed once with PBS. Lysis was achieved by incubating the cells in the recommended volume of freshly prepared 1× *Renilla* Luciferase lysis buffer for the culture vessel. Lysates were then stored at −20 °C until assayed for RLuc activity as half-scale reactions. Luciferase assays were performed in white LUMITRAC plates (Greiner Bio-one, Kremsmünster, Austria) using a GloMAX^®^ Explorer microplate reader (Promega).

### 2.11. Replicon Antiviral Assay

Replicon transfected cells were seeded into 96-well plates at a density of 10,000 cells per well; 2× concentrated serial dilutions of the indicated compounds remdesivir (Cambridge Bioscience, Cambridge, UK), ritonavir (Sigma-Aldrich, Merck, Darmstadt, Germany) or cobicistat (Santa Cruz Biotechnology, Inc., Dallas, TX, USA) were then added immediately upon cell seeding and diluted to the final desired 1× concentration directly in each well. At 24 hpt, the cells were harvested for luciferase assays as described, and percentage inhibition relative to the vehicle-only treatment was determined.

### 2.12. Rescue of Recombinant SARS-CoV-2

The respective BAC templates used to produce recombinant (r)SARS-CoV-2 were pSC2-S:D614G, pSC2-nsp1:KH-AA, pSC2-ORF6:mS [51] and pSC2-S:Delta (Table S6). To rescue recombinant virus, BAN cells were first transfected with 10 µg capped in vitro RNA transcripts as described above. The cells were then incubated for 3 days or until extensive cytopathic effect (CPE) was observed. Supernatants were then filtered through a 0.22 µM filter and stored at −80 °C as the P0 stock.

### 2.13. Viral Stock Preparation

Stocks of an early SARS-CoV-2 B.1 lineage clinical isolate (REMRQ0001) isolated in April 2020 as previously described [52] and rSARS-CoV-2 viruses were produced by infecting VTN or VAT cells at 75–90% confluence with an approximate multiplicity of infection (MOI) of 0.0001–0.01 of virus in MEM supplemented with 1 mM sodium pyruvate, 2% (*v*/*v*) FBS and 0.1 mM non-essential amino acids (MEM 2%FBS). Flasks were then incubated at 37 °C until the desired amounts of CPE were reached. The supernatant was then collected, buffered to 25 mM HEPES, pH 7.0, filtered through a 0.22 µM filter and stored at −80 °C.

### 2.14. Immunofluorescence Assay

Cells transfected with replicon in vitro RNA transcripts or to be infected with recombinant virus or mock infected were seeded in appropriate media in µClear 96-well Microplates (Greiner Bio-one) and grown at 37 °C in 5% CO_2_. After an appropriate incubation/infection period, the cells were fixed in 4% paraformaldehyde (*v*/*v*) in PBS for 10 min. Fixed cells were permeabilized with 0.1% (*v*/*v*) Triton-X100 in PBS and blocked with 1% (*w*/*v*) bovine serum albumin before staining with a monoclonal antibody against either the SARS-CoV-2 N protein (1:1000 dilution; 200-401-A50, Rockland, Baltimore, MD, USA), double-stranded RNA (dsRNA) (1:250 dilution, J2 10010200, Scicons, Nordic MUbio, Susteren, Netherlands) or eGFP (1:500 dilution, AB0020-200, SICGEN, Cantanhede, Portugal) followed by an appropriate Alexa Fluor-conjugated secondary antibody (1:3000 dilution, Invitrogen™, Thermo Fisher Scientific) and 4′,6-diamidino-2-phenylindole (DAPI)/Hoechst 33342 (Sigma Aldrich). To determine the number of replicon-expressing cells, images were acquired on an ImageXpress Pico Automated Cell Imaging System (Molecular Devices, San Jose, CA, USA) using a 10× objective. Stitched images of 9 fields covering the central 50% of the well were analysed using Cell Reporter Xpress software (Version 2.9, Molecular Devices, San Jose, CA, USA).

### 2.15. Viral Stock Titration

Virus was titred by half-dilutions across µClear 96-well Microplates in technical triplicates. Virus added to well 1 was added neat or diluted up to 1:10 *v*/*v* based on expected viral titres. After 8 h the number of infected cells was determined by immunofluorescence assay and automated image analysis and the infectious titre determined in the linear part of the titration curve.

### 2.16. Viral Growth Analysis

VTN, AAT or BEAS-2BA cells were seeded at 50,000 cells per well in 24-well plates one day prior to infection. For Calu-3 ALI cultures, a culture insert was dissociated, and cells counted to determine cell number and MOI. The cell cultures were then infected at an MOI of 0.01 in a total volume of 0.25 mL at 37 °C for 2 h, rocking the plates approximately every 20 min. After infection the viral inoculum was removed, and the cell layers were washed with DPBS. For submerged cultures, 1 mL of MEM 2%FBS was then added per well and 0.6 mL of supernatant sampled and replaced at 4, 8, 24, 48 and 72 h post-infection (hpi). For ALI cultures, released infectious virus was collected in 200 µL MEM 2%FBS by adding it to the apical culture side and allowing virus to diffuse into the supernatant for 15 min, after which it was removed and titrated. The amount of infectious virus released into the culture supernatant was then determined by titration on VTN cells as described above.

### 2.17. Methylthiazolyldiphenyl-Tetrazolium Bromide (MTT) Cell Viability Assay

To assess the viability of mock and infected VTN and AAT cells at desired time-points, infection media was removed from the cells and replaced with 1 mg/mL MTT (Sigma-Aldrich, Merck, Darmstadt, Germany) in DPBS with further incubation for 3 h at 37 °C. The MTT reagent was then removed, and the cells solubilised with absolute ethanol. Colorimetric measurement was performed using the GloMax^®^ Explorer (Promega) at 600 nm wavelength. Data were then normalised to mock and analysed.

### 2.18. Interferon Dose–Response Assays

VTN or AAT cells were seeded at 10,000 cells per 96-well one day prior to infection. The cells were then pre-treated with a five-point 10-fold dilution series of universal IFN (IFN-α, PBL Assay Science, Piscataway, NJ, USA) starting at 1000 U/mL. On the day of infection, the pre-treatment media was removed, and the cells were washed with Dulbecco’s PBS (DPBS). The cells were then infected at MOI 0.01 for 1 h and the inoculum removed. The cells were then incubated with MEM 2%FBS without IFN for 24 h and viral growth was assessed by immunofluorescence assay.

### 2.19. Single-Molecule Fluorescence In Situ Hybridisation (smFISH) of Replicon RNA

smFISH was carried out as previously reported [53]. Briefly, VTN cells transfected with replicon in vitro RNA transcripts were grown on #1.5 round-glass coverslips in a 24-well plate, fixed in 4% paraformaldehyde (Thermo Fisher Scientific) for 30 min at room temperature. Cells were permeabilised in DPBS/0.1% Triton X-100 for 10 min at room temperature followed by two washes in DPBS and 2× SSC. Cells were pre-hybridised in pre-warmed (37 °C) wash solution (2× SSC, 10% formamide) twice for 20 min at 37 °C. smFISH probes specific for the SARS-CoV-2 ORF1a region were diluted to 500 nM in hybridisation solution (2× SSC, 10% formamide, 10% dextran sulphate) and incubated overnight at 37 °C. Coverslips were then washed for 20 min in pre-warmed wash solution at 37 °C followed by counterstain with DAPI (1 μg/mL) and CellMask Green (Invitrogen, Thermo Fisher Scientific; 1:1,000,000) diluted in wash solution. Post staining cells were washed once with wash solution for 20 min at 37 °C and twice with 2× SSC for 10 min at room temperature. Coverslips were mounted using Vectashield onto glass slides and imaged on an Olympus SoRA Super-res confocal system. Automatic and manual image acquisition and image stitching were performed with Olympus cellSens Dimension software, (Version 4.3). Images were analysed with a custom Python (Version 3.7) pipeline using Bigfish, skimage, and numpy libraries (available in the GitHub repository https://github.com/jefflee1103/Lee_Wing-SARS2/tree/main/smFISH/analaysis-codes/Bigfish-pipeline, accessed on 1 October 2023). TIFF files were converted to a numpy array, and individual cells segmented from the image using the Cellpose library. Background signal in the smFISH channel was subtracted with the skimage.white_tophat algorithm (radius = 5, individual zframes were processed in 2D due to memory constraints, results were indistinguishable from 3D-processed images). Threshold setting for smFISH spot detection was set specifically for each set of images collected in each session. Replication foci were defined as genomic RNA (gRNA) smFISH signals with spatially extended foci that exceed the point-spread function of the microscope and intensity of the reference single molecules.

### 2.20. Ruxolitinib Dose–Response Assays

AAT cells were seeded at 10,000 cells per 96-well one day prior to infection. The cells were then pre-treated with a serial dilution of ruxolitinib (Cell Guidance Systems, Cambridge, UK) for 2 h. The cells were then infected at MOI 0.025 for 1 h and the inoculum removed. The cells were then washed and incubated with MEM 2%FBS containing ruxolitinib. At 24 hpi the cells were fixed, and relative infection assessed by immunofluorescence assay.

## 3. Results

A number of sub-genomic SARS-CoV-2 replicon cDNA clones were designed and tested during this investigation, with the overall aim of producing an RNA launched replicon that could be stably maintained in cells. Unlike previous studies, our work included replicons derived not only from the ancestral B.1 isolate (Wuhan-Hu-1) but also from the Delta VOC. In both cases, multiple viral coding regions were replaced to enable sensitive and versatile readouts of replicon replication, including fluorescence (mNeonGreen and mScarlet) and *Renilla* luciferase (RLuc) activity. To reduce cytotoxic effects associated with replicon expression, targeted mutations were introduced into the nsp1 gene. Additionally, all replicons were engineered to express the *pac* gene product, enabling the selection of cells that stably maintain replicons under puromycin treatment.

### 3.1. Construction of a First-Generation Wuhan-Hu-1 SARS-CoV-2 Replicon

To assemble the first-generation SARS-CoV-2 replicon, nine cDNA fragments spanning the entire SARS-CoV-2 isolate Wuhan-Hu-1 genome, except for the S and M gene coding sequences, were assembled into the pYES1L BAC/YAC shuttle vector using TAR assembly in yeast to produce clone pSC2-Rep-Gp-RL. The S and M gene coding sequences were replaced with sequences encoding an eGFP-pac fusion protein and RLuc, respectively (Figure 1). Bacmid sequencing verified the introduced changes. Previously, TAR has been used in the assembly of recombinant CoV genomes and replicons, including the first recombinant version of SARS-CoV-2 [16,32,51,54,55]. Mutations were introduced into the 5′-terminal cDNA fragment, encoding the nsp1 amino acid substitutions K164A/H165A (nsp1:KH-AA), to reduce cytotoxic effects induced by replicon replication. These mutations were also introduced into an infectious recombinant SARS-CoV-2 clone, pSC2-nsp1:KH-AA, to study any potential effects of these mutations on virus replication.

**Figure 2:**
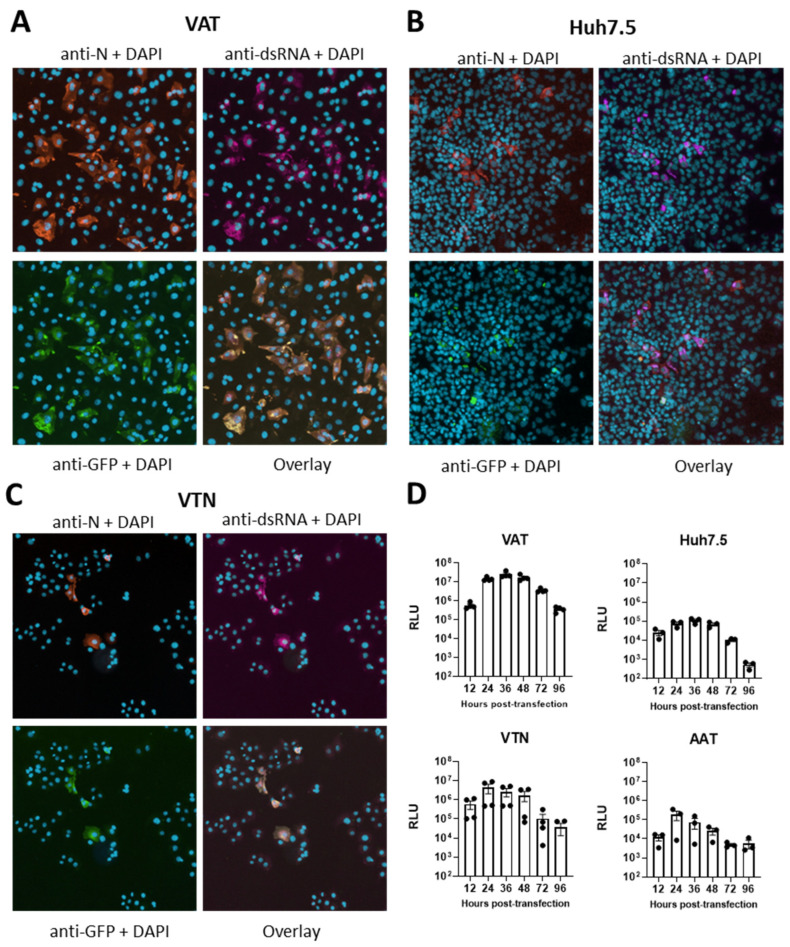
Characterisation of replicon Rep-Gp-RL replication (**A**–**C**). Immunofluorescence assay of replicon transfected cells. (**A**) VAT, (**B**) Huh 7.5 and (**C**) VTN cells were transfected with in vitro RNA transcripts corresponding to Rep-Gp-RL and N gene constructs. Cells shown in the top panels were stained with antibodies recognising the SARS-CoV-2 N protein and dsRNA with nuclear DNA identified by DAPI staining. Bottom panels show staining with an antibody recognising eGFP, nuclear DNA stained with DAPI and a four-colour overlay. (**D**) VAT, Huh 7.5, VTN and AAT cells were transfected with Rep-Gp-RL RNA transcripts and N gene constructs. At the indicated times post-transfection, the cells were lysed, and the lysates assayed for RLuc activity. Luminescence of each sample was adjusted for background by subtracting a cell-only control. Graphs show the mean and standard error of the mean (SEM) of n = 3–4 biological repeats.

In vitro RNA transcripts were prepared from the linearised pSC2-Rep-Gp-RL bacmid, corresponding to the replicon Rep-Gp-RL and transfected into VAT, Huh 7.5, VTN and AAT cells. RNA transcripts were capable of initiating replication in all cell types, as determined by immunofluorescence staining for the viral replication marker double-stranded (ds)RNA [7,56] (Figure 2A–C) and the detection of RLuc activity (Figure 2D). Although RLuc activity was readily detected, eGFP fluorescence was not. To determine whether a transcript capable of producing eGFP-pac was produced, transfected cells were stained with an anti-eGFP antibody (Figure 2A–C). Staining confirmed co-localisation of dsRNA and eGFP, suggesting either production of a non-fluorescent protein or production of eGFP at a level that could not be detected (Figure 2A–C). Interestingly, a sequence-identical eGFP-pac gene was previously used to produce puromycin resistant and fluorescent cells that stably express a dengue virus replicon [57].

RLuc was detected at low levels as early as 12 hpt in the cell types tested. The kinetics of RLuc expression were similar in the four cell types with maximal RLuc activity observed between 24 and 36 hpt over a 96 h period (Figure 2D). However, the levels of RLuc activity were markedly different in the VAT cells (peak of 2.5 × 10^7^ relative luminescence units (RLUs)) compared to the other three cell types (peaks of 1.5–3 × 10^5^ RLU). The increased levels of RLuc activity in VAT cells corresponded to an increased transfection efficiency (~20%) compared to the other cells tested in this study (Figure 2A–C and Figure S1) and reported in other studies [28,32]. As such, the VAT cell line was routinely used in this study for testing other replicon constructs and antiviral assays.

**Figure 3:**
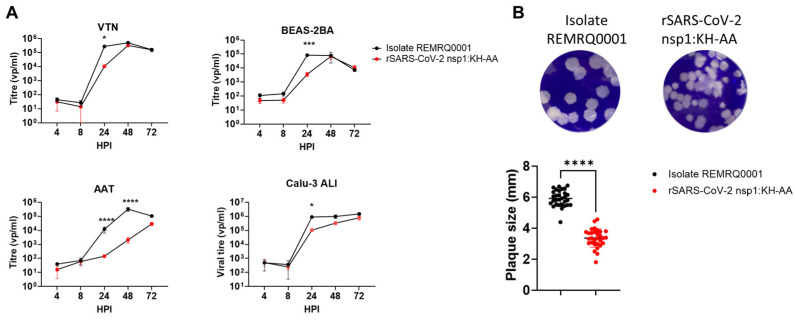
(**A**) Replication kinetics of rSARS-CoV-2 nsp1:KH-AA and wild-type SARS-CoV-2 isolate REMRQ0001 in VTN, BEAS-2BA, AAT and Calu-3 cells. Graphs show mean and SEM of n = 3 biological repeats. Statistical analysis was performed using two-Way ANOVA with * < 0.05, *** < 0.0002 and **** < 0.0001. (**B**) Plaque morphology of rSARS-CoV-2 nsp1-KH-AA and REMRQ0001 in VTN cells. Representative image of plaques formed by each virus. Using Fiji, the diameter of 10 plaques in three biological repeats was quantified. An unpaired *t*-test was performed, with *p* < 0.05 shown as *.

To assess whether replicon replication was affected by the introduced nsp1:KH-AA mutation, the impact of this mutation was investigated in the context of an infectious rSARS-CoV-2; rSARS-CoV-2 nsp1:KH-AA. The replication of rSARS-CoV-2 nsp1:KH-AA and a B.1 lineage virus isolate (REMRQ0001), both of which lacked the S protein D614G mutation, was assessed in VTN, BEAS-2BA, AAT and Calu-3 cells (Figure 3A). In VTN, BEAS-2BA and Calu-3 cells rSARS-CoV-2 nsp1:KH-AA was found to have a significant reduction in viral titres at 24 hpi but was largely comparable in titre to the wild-type REMRQ0001 isolate at other time-points post-infection. However, rSARS-CoV-2 nsp1:KH-AA showed attenuated growth in AAT cells at 24- and 48-hpi. No significant difference in viral titres could be detected 72 hpi. In addition to a slight growth attenuation in VTN cells, the plaque size of rSARS-CoV-2 nsp1-KH-AA was also significantly reduced compared to REMRQ0001 (Figure 3B). Regardless, the nsp1:KH-AA mutations were stably retained in the viral genome over five sequential passages in either VTN or AAT cells.

**Figure 4:**
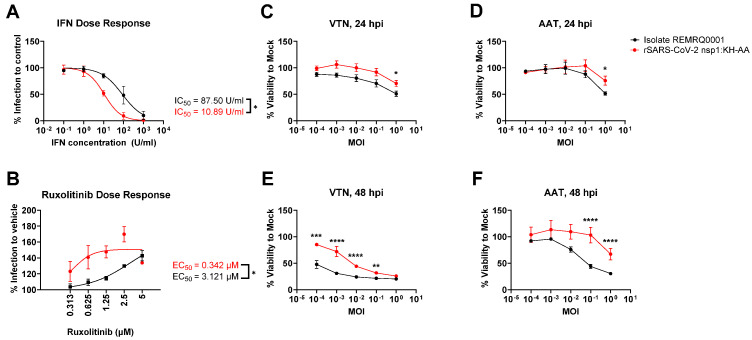
(**A**) Dose response assay of rSARS-CoV-2 nsp1:KH-AA and SARS-CoV-2 REMRQ0001 to IFN-α. VTN cells were pre-treated for 18 h with the indicated concentrations. Cells were then washed before infection at MOI 0.01, washed and incubated in infection media. At 24 hpi, cells were fixed and relative infection was determined by immunofluorescence staining of N and DAPI staining. The graph shows the mean and SEM of n = 3 biological replicates. (**B**) Drug response assay of rSARS-CoV-2 nsp1:KH-AA and REMRQ0001 to the JAK-STAT pathway inhibitor ruxolitinib. AAT cells were pre-treated for 2 h with the indicated concentrations. Cells were then infected at MOI 0.025, washed and incubated with the indicated concentration for 24 h. Relative infection was determined by immunofluorescence of N and DAPI staining. The graph shows the mean and SEM of n = 3 biological replicates. Statistical analysis comparing REMRQ0001 vs. rSARS-CoV-2 nsp1:KH-AA was performed using a two-way ANOVA, with significance taken as *p*-value < 0.05, represented as *. (**C**–**F**) Cellular viability assay in VTN and AAT cells comparing REMRQ0001 and rSARS-CoV-2 nsp1:KH-AA. VTN or AAT cells were infected at the indicated MOIs and incubated for 24- or 48-hpi. At each time-point, an MTT assay was performed to determine the viability of the infected cultures, normalising cell viability to the uninfected control. Statistical analysis comparing REMRQ0001 vs. rSARS-CoV-2 nsp1:KH-AA was performed using a two-way ANOVA, with significance taken as *p*-value < 0.05, represented as ** = 0.0098, *** = 0.0001, **** < 0.0001.

rSARS-CoV-2 nsp1:KH-AA was found to be more attenuated in BEAS-2BA and AAT cells compared to REMRQ0001. Both cell lines have a competent IFN response unlike VTN cells [58] (Figure 3A). Furthermore, Rep-Gp-RL also replicated less well in Huh 7.5 and AAT cells when compared to VAT and VTN cells (Figure 2D). Huh 7.5 cells have a deletion in retinoic acid-inducible gene I (RIG-I), rendering the pathway non-functional, but may mount an interferon response via alternative sensors of viral infection [48,59]. To investigate whether the nsp1:KH-AA mutation sensitised the recombinant virus to IFN, virus replication studies were performed using VTN cells pre-treated with exogenous IFN-α (Figure 4A). Compared to REMRQ0001, the replication of rSARS-CoV-2 nsp1:KH-AA in VTNs was found to be more strongly restricted by pre-treatment with IFN-α with respective half maximal inhibitory concentration (IC_50_) values of 87.50 U/mL and 10.89 U/mL. To investigate whether the replication of rSARS-CoV-2 nsp1:KH-AA could be rescued in an IFN competent cell line, AAT cells were treated pre- and post-infection with ruxolitinib, an inhibitor of the Janus kinase-signal transducer and activator of transcription (JAK-STAT) pathway. Treatment of AAT cells with ruxolitinib resulted in increased replication of both viruses, with a more pronounced effect on the replication of rSARS-CoV-2 nsp1:KH-AA (Figure 4B).

The effects of the nsp1:KH-AA mutation on the cellular toxicity of SARS-CoV-2 replication were then investigated, as previously the mutations were introduced into SARS-CoV-2 replicons to potentially reduce CPE and allow stable cell line generation [32,46]. VTN and AAT cells were infected with ten-fold dilutions of rSARS-CoV-2 nsp1:KH-AA and REMRQ0001. At 24- and 48 hpi, the cellular viability was determined by MTT assay (Figure 4C–F). The MTT assay showed that infection with rSARS-CoV-2 nsp1:KH-AA resulted in a minor but significant increase in cell viability at 24 hpi compared to infection with REMRQ0001 (Figure 4C,D), with a more pronounced increase in viability by 48 hpi (Figure 4E,F).

### 3.2. Construction of Second-Generation Wuhan-Hu-1 SARS-CoV-2 Dual-Reporter Replicons

**Figure 5:**
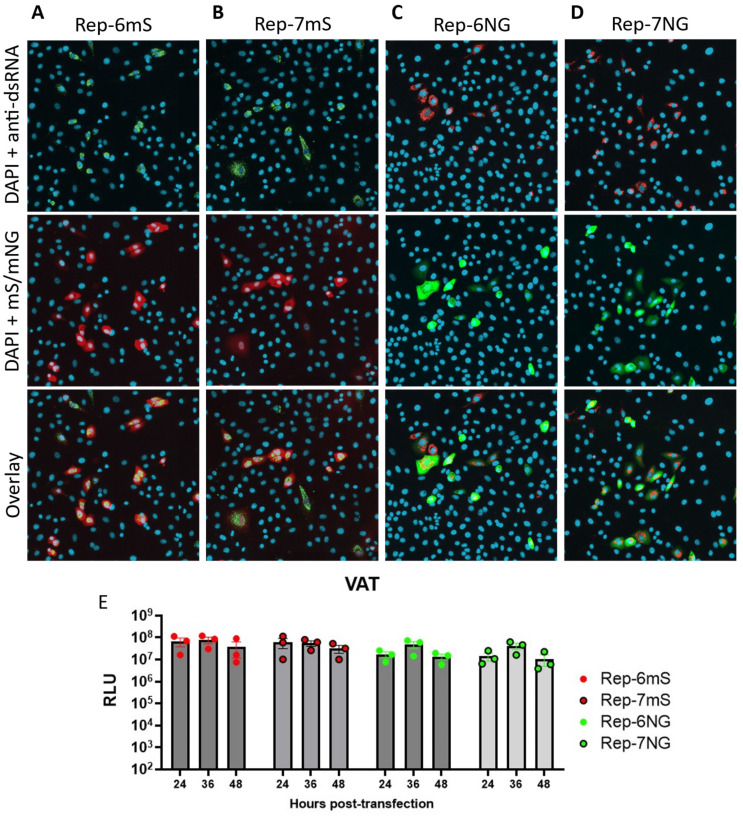
Characterisation of second-generation SARS-CoV-2 replicons. (**A**–**D**) Immunofluorescence assay of replicon transfected cells. Detection of mScarlet, mNeonGreen and dsRNA after transfection of in vitro RNA transcripts derived from dual reporter replicons. VAT cells were transfected with in vitro RNA transcripts corresponding to (**A**) Rep-6mS, (**B**) Rep-7mS, (**C**) Rep-6NG and (**D**) Rep-7NG and an N gene construct. Cells in the top panels were stained for dsRNA and nuclear DNA (DAPI). The middle panels show fluorescence from the mScarlet (mS) and mNeonGreen (mNG) proteins and DAPI staining of nuclear DNA, whilst the bottom panels show a three-colour overlay. Fluorescence from the reporter proteins was acquired using an ImageExpressPico microscope. (**E**) Luciferase kinetics of second-generation replicons. Rluc activity after transfection of in vitro RNA transcripts derived from dual reporter replicons. VAT cells were transfected with in vitro RNA transcripts corresponding to Rep-6mS, Rep-7mS, Rep-6NG, Rep-7NG and an N gene construct. At the indicated times post-transfection, the cells were assayed for RLuc activity. The sample luminescence was adjusted for assay background by subtracting the cell-only control. Graphs show mean and SEM of n = 3 biological replicates.

To produce a functional dual reporter replicon, pSC2-Rep-Gp-RL was further modified. The S gene coding sequence was replaced with a codon-optimised *pac* gene sequence. Furthermore, to produce replicons that could express a fluorescent marker protein, either the ORF6 or ORF7a coding sequences were replaced with mNeonGreen [60] or mScarlet [61] coding sequences (Figure 1B, Table S5). The ORF6 TRS is located within the coding sequence of the M gene [2], which was replaced with the RLuc coding sequence, and therefore the TRS was faithfully re-introduced upstream of the ORF6 gene replacement during the cloning process. In addition, the 3′-terminus of the replicon plasmids was modified to introduce a hepatitis delta virus ribozyme sequence and T7 RNA polymerase terminator sequence immediately downstream of the encoded poly-A tail (Table S2), removing the need to linearise the replicon plasmids prior to in vitro transcription. The resulting clones were termed pSC2-Rep-p-RL-6NG, pSC2-Rep-p-RL-6Scar, pSC2-Rep-p-RL-7NG and pSC2-Rep-p-RL-7Scar (Figure 1B) with the replicons corresponding to the clones termed Rep-6NG, Rep-6mS, Rep-7NG and Rep-7mS, respectively. To assess expression of the fluorescent reporter proteins and RLuc in vitro, RNA transcripts corresponding to the replicons were transfected into VAT cells. All four replicons were found to be replication competent, as determined by the identification of dsRNA and the expression of mNeonGreen or mScarlet from sgRNA transcripts (Figure 5A–D). RLuc activity was assessed at 24-, 36- and 48-hpt (Figure 5E and Figure S2). The replication kinetics of the different replicons was similar to Rep-Gp-RL (Figure 2D), with maximal expression at 36 hpt in VAT cells. The level of peak expression was also of a similar magnitude as before (ranging from 1.8 to 4.5 × 10^7^ RLU). There were some differences in RLuc expression resulting from transfection of the different replicon in vitro RNA transcripts, which could have been due to differences in the quality of the transcripts, the transfection efficiency or expression levels from the ORF6 or ORF7 sgRNAs encoding the mNeonGreen/mScarlet fluorophores. As for Rep-Gp-RL, numerous attempts were made to isolate cells stably harbouring all four of the dual reporter replicons under puromycin selection using a range of cell types (Table S9). However, stable cell line selection was not possible, suggesting that replicon replication was still cytotoxic despite the presence of the nsp1 mutations.

### 3.3. Construction of a Dual-Reporter Replicon for the SARS-CoV-2 Delta VOC

**Figure 6:**
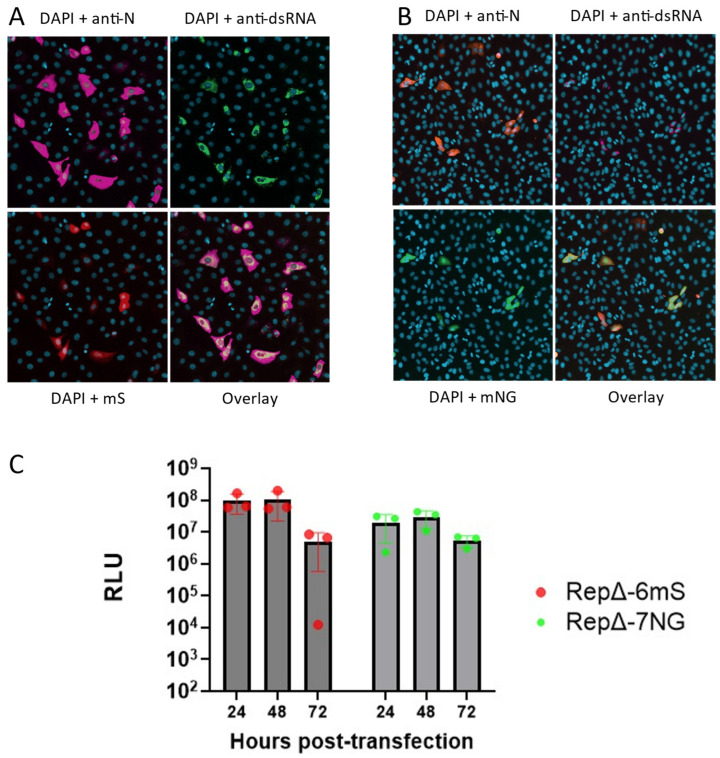
Validation of the replication of in vitro RNA transcripts derived from SARS-CoV-2 Delta VOC replicons. (**A**,**B**) Immunofluorescence assay of replicon transfected cells. VAT cells were transfected with in vitro RNA transcripts corresponding to (**A**) RepΔ-6mS and (**B**) RepΔ-7NG, respectively, and an N gene construct. At 24 hpt, a portion of the cells was fixed and stained with antibodies recognising the N protein and dsRNA and examined for fluorescence from (**A**) mScarlet, (**B**) mNeonGreen and DAPI stained nuclear DNA. The bottom right panels show a three-colour overlay. Fluorescence from the reporter proteins was acquired using an ImageExpressPico microscope. (**C**) Luciferase kinetics of second-generation Delta replicons. Expression of RLuc in VAT cells transfected with in vitro RNA transcripts corresponding to RepΔ-6mS and RepΔ-7NG was monitored over the period of 24–72 hpt. The sample luminescence was adjusted for assay background by subtracting the cell-only control. Graphs show mean and SEM of n = 3 biological repeats.

As the SARS-CoV-2 pandemic has progressed, new VOCs have emerged, exhibiting numerous lineage-defining mutations that necessitate further investigation. Consequently, a replicon corresponding to the SARS-CoV-2 Delta VOC was generated. The same design as for the dual reporter replicons Rep-6mS and Rep-7NG was adopted given they mediated expression of RLuc and mScarlet/mNeonGreen (Figure 5). Lineage-defining mutations characteristic of the SARS-CoV-2 Delta VOC were introduced into the replicon BAC/YAC clones during the assembly process by substitution of cDNA fragments corresponding to the Wuhan sequence with those produced by RT-PCR amplification from SARS-CoV-2 Delta VOC genomic RNA (shown in Table S4). In addition, OL-PCR and Hi-Fi assembly were used to clone the mNeonGreen/mScarlet reporter genes into the Delta VOC genetic background. The fragments were then assembled into BAC/YAC plasmids to produce the replicon constructs pSC2-Rep-Del-p-RL-6Scar and pSC2-Rep-Del-p-RL-7NG with the corresponding replicons termed RepΔ-6mS and RepΔ-7NG respectively. VAT cells were transfected with in vitro RNA transcripts corresponding to RepΔ-6mS and RepΔ-7NG to test their functionality. Both replicons were capable of mediating replication, as evidenced by the presence of dsRNA and the expression of RLuc and the fluorescent proteins from sgRNAs (Figure 6).

### 3.4. Comparison of Replicon and Virus RNA Replication

**Figure 7:**
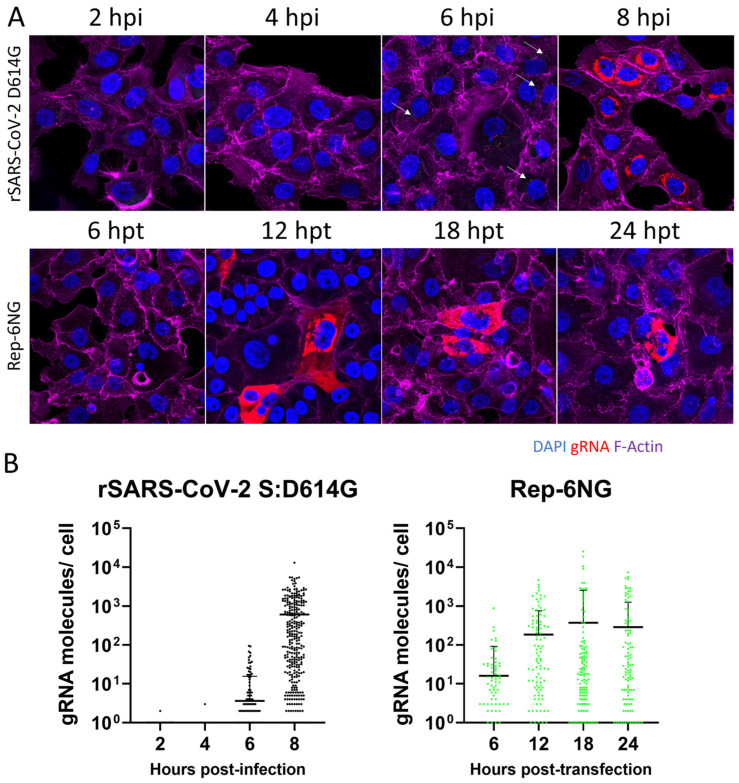
smFISH RNA copy kinetics of rSARS-CoV-2 S:D614G and Rep-6NG in VTN cells. (**A**) VTN cells were grown on coverslips and infected at MOI 3 with rSARS-CoV-2 S:D614G or transfected with 10 µg in vitro RNA transcripts corresponding to Rep-6NG as described and seeded onto coverslips. The image shows representative infected/transfected cells fixed at the selected hpi/hpt, respectively, and stained for F-actin, gRNA and nuclear DNA. White arrows indicate viral gRNA (**B**). Quantification of SARS-CoV-2 viral and replicon gRNA molecules/cell was performed using FISH-quant/big-fish and data points show the average count of 5–10 fields of view, with 3–5 cells sampled/area.

To investigate and compare the replication of the replicons to live virus, the genome copies of rSARS-CoV-2 S:D614G and Rep-6NG were quantified using smFISH as described previously [53,62]. VTN cells were either infected at MOI 3 or transfected with 10 µg of in vitro RNA transcripts corresponding to Rep-6NG and 2 µg N-gene transcripts and fixed at 2, 4, 6, 8 and 6, 12, 18 and 24 hpi/hpt, respectively. The amount of genomic RNA (gRNA) was then analysed and subsequently quantified using smFISH (Figure 7).

Imaging of the smFISH stained samples indicated potential differences between virus and replicon (Figure 7A). The localisation of gRNA in virally infected cells appeared to be tightly associated with the perinuclear region whereas the replicon-expressing cells showed distribution of gRNA across the whole cytoplasm at later time-points. Quantification of viral and replicon gRNA copies revealed differences in their RNA copy kinetics (Figure 7B). An observation period of 8 h (virally infected cells) and 24 h (replicon transfected) was chosen to represent one round of viral replication (entry to release) and one cell division for replicon transfected cells. The viral gRNA could be detected at low abundance (1–2 genomes/cell) at 2- and 4-hpi. This may correspond to the incoming viral genome [53]. Viral gRNA copy numbers started to exponentially increase after 4 hpi and again from 6 to 8 hpi. The replicon transfected cells at 6 hpt showed a higher amount of gRNA copies than the virus. However, in contrast to the virus, the replicon gRNA copies gradually increased to reach their peak at 18 hpt. After 18 h, the gRNA copy number remained stable with a trend towards a reduction in gRNA copies after 24 h.

### 3.5. Replicons Show Comparable Response to Remdesivir Compared to Wild-Type Virus

**Figure 8:**
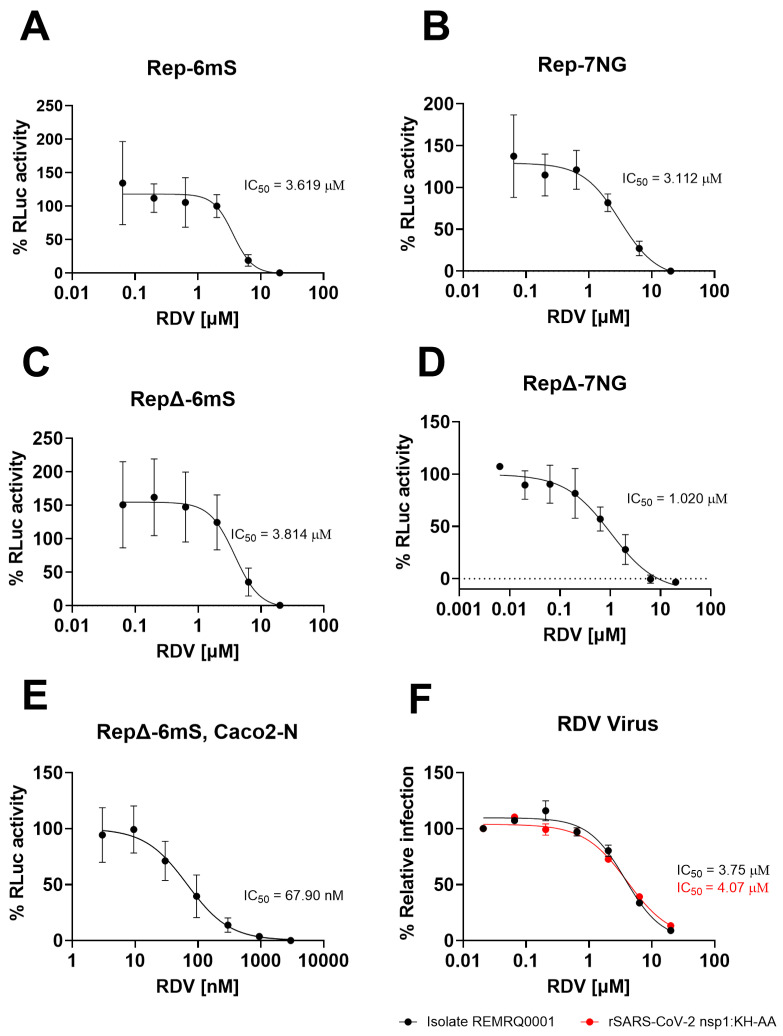
Replicons and rSARS-CoV-2 nsp1:KH-AA are dose-responsive to remdesivir. (**A**–**D**) Replicon dose–response curves to remdesivir. VAT cells were transfected with in vitro RNA transcripts corresponding to (**A**) Rep-6mS, (**B**) Rep-7NG, (**C**) RepΔ-6mS and (**D**) RepΔ-7NG and an N gene transcript. (**E**) Caco2-N cells were transfected with in vitro RNA transcripts corresponding to RepΔ-6mS and an N gene transcript. The transfected cells were incubated with a half-log fold dilution series of remdesivir, starting at 20 µM. At 24 hpt the cells were lysed and assayed for RLuc activity. The graphs show mean and standard deviation as a percentage of the dimethyl sulfoxide (DMSO) control, adjusted for background. (**F**) Dose–response curve of REMRQ0001 and rSARS-CoV-2 nsp1:KH-AA to remdesivir. VTN cells were infected with REMRQ0001 and rSARS-CoV-2 nsp1-KH-AA at MOI 0.5. The infected cells were incubated for 18 h with a half-log dilution series of remdesivir starting at 20 μM. Relative infection was determined by immunofluorescence and expressed as a percentage of the DMSO vehicle. IC_50_ values were determined in GraphPad Prism (Version 10.2.3, Boston, MA, USA).

Having established that the produced replicons are capable of faithfully recapitulating intracellular SARS-CoV-2 replication, their use as an antiviral screening tool was evaluated. The replicons Rep-6mS, Rep-7NG, RepΔ-6Scar and RepΔ-7NG were tested for their sensitivity to remdesivir using VAT cells (Figure 8A–D and Figure S3). To test this system in human cell lines, Caco2-N cells, which had a similar kinetics of replicon replication to VAT cells (Figure S4), were transfected with in vitro RNA transcripts corresponding to RepΔ-6mS (Figure 8E and Figure S5). The replicon transfected cells were seeded into 96-well plates and then incubated with a half-log 8-point dilution series of remdesivir starting with 20 µM as the highest dose. At 24 hpt, prior to the peak of maximal RLuc activity in previous assays, the cells were harvested, and lysates assayed for RLuc activity. All replicons demonstrated a dose responsive reduction in RLuc activity with increasing doses of remdesivir (Figure 8A–D and Figure S3). This confirmed that the luciferase reporter, translated from the sub-genomic RNA produced from the M-TRS, can be used as a surrogate read-out for authentic viral replication in a variety of cell lines. IC_50_ values for Rep-6mS and Rep-7NG were 3.62 µM and 3.11 µM, respectively. The Delta VOC replicons were determined to have IC_50_ values for RepΔ-6mS and RepΔ-7NG as 3.81 µM and 1.02 µM, respectively. Vero cells express efflux pumps and therefore IC_50_ values in these cells are often higher than in human cell lines [63]. As expected, remdesivir showed heightened potency against the replication of RepΔ-6mS in a human cell line, Caco2-N, with an IC_50_ value of 67.9 nM compared to 3.81 µM in VAT cells.

To compare these values to infectious virus, the clinical isolate REMRQ0001 and parental rSARS-CoV-2 nsp1:KH-AA viruses were titrated against remdesivir. VTN cells were infected at MOI 0.5 and the cells incubated for 18 h with half-log dilutions of remdesivir, starting at 20 µM. Using this assay, IC_50_ values of 3.75 and 4.07 µM were calculated, respectively, corresponding closely to the tested replicons. These values are in the range of IC_50_ values obtained for remdesivir using assays based on SARS-CoV-2 infection of Vero cells and other SARS-CoV-2 replicon studies using Vero cells [28,64]. The results demonstrate that the replicons provide a valuable platform for antiviral testing. As the replicons behaved comparably, Rep-6mS and RepΔ-6mS were arbitrarily carried forward for further antiviral testing.

### 3.6. Replicons of Wuhan and Delta Lineage Show Differential Drug Responses to Ritonavir and Cobicistat

After establishing the replicons can be used as a surrogate for viral replication, two further compounds were tested for their antiviral properties. Cobicistat and ritonavir are structurally related compounds that target cytochrome P450 3A class molecules and P-glycoprotein [65]. Cobicistat has been shown to inhibit the replication of the SARS-CoV-2 Wuhan isolate, by preventing S protein-mediated fusion and viral entry [66]. Quantitative RT-PCR analysis of intracellular viral RNA in cobicistat treated cells revealed a decrease in overall virus replication, suggesting that cobicistat may also inhibit intracellular RNA replication. To assess the impact of both cobicistat and ritonavir on the intracellular replication of SARS-CoV-2, VAT cells were transfected with 2 µg N-gene transcripts and 10 µg of in vitro RNA transcripts corresponding to Rep-6mS or RepΔ-6mS. The cells were then incubated with five-point two-fold dilutions of each drug, starting at 20 µM, a concentration we previously showed was well tolerated by Vero E6 cells [67]. At 24 hpt, the cells were lysed and assayed for RLuc activity.

**Figure 9:**
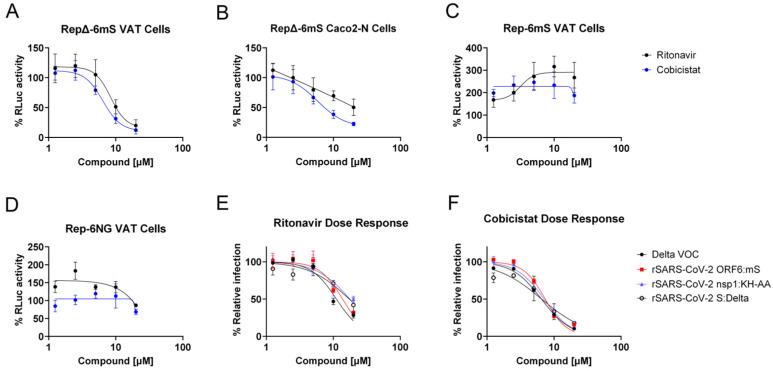
Dose–response assays with ritonavir and cobicistat. VAT (**A**,**C**,**D**) and Caco2- N cells (**B**) were transfected with 2 µg of N-gene transcripts and 10 µg replicon transcripts as indicated. Cells were then seeded and incubated with a two-fold five-point dilution series of drug, starting at 20 µM. At 24 hpt, the cells were lysed and analysed for RLuc activity. The RLuc activity was then expressed as a percentage of the DMSO vehicle control. The graphs show mean and standard deviation of n = 3–4 biological repeats. (**E**,**F**) VTN cells were infected with the indicated SARS-CoV-2 viruses at MOI 0.5. The infected cells were incubated for 18 h with two-fold dilution series of each drug starting at 20 μM. Relative infection was determined by immunofluorescence and expressed as a percentage of the DMSO vehicle. IC_50_ values were determined in GraphPad Prism (Version 10.2.3, Boston, MA, USA).

Intracellular replication of the RepΔ-6mS replicon was inhibited by ritonavir and cobicistat, with IC_50_ values of 8.42 and 6.44 µM, respectively (Figure 9A). This inhibitory effect was also observed in human Caco2-N cells (Figure 9B). By contrast, replication of Rep-6mS and Rep-6NG was not markedly inhibited by either compound. Instead, replication appeared to slightly increase in the presence of ritonavir for Rep-6mS and Rep-6NG (Figure 9B,C). Interestingly we previously found that the replication of both the wild-type Wuhan and Delta viruses in Vero E6 cells was inhibited by both compounds with IC_50_ values of 9.5 µM (Wuhan) and 6.8 µM (Delta) for cobicistat and 17.1 µM (Wuhan) and 8.9 µM (Delta) for ritonavir [67]. To further investigate this differential response between the Wuhan Hu-1 and Delta VOC replicons, an antiviral assay using rSARS-CoV-2 viruses was performed. The viruses used were the replicon parental viruses rSARS-CoV-2 nsp1:KH-AA and the Delta VOC and rSARS-CoV-2 in which ORF6 was replaced with mScarlet (rSARS-CoV-2 ORF6:mS) and S was replaced with the Delta VOC S (rSARS-CoV-2 S:Delta). All viruses were found to be effectively inhibited by both compounds in a dose-responsive manner. The IC_50_ values for ritonavir were calculated as 11.15, 13.75, 17.73 and 17.34 µM, respectively (Figure 9E). The IC_50_ values for cobicistat were calculated as 6.49, 7.40, 7.10 and 6.53 µM, respectively (Figure 9F). Together, these data suggest the main activity mechanism of the drugs is exerted by interfering with S-mediated entry, or in a process during infection that is not re-capitulated in the Wuhan Hu-1 replicons. However, the data also suggest that a Delta VOC viral protein, or Delta VOC specific host factor, is targeted by the drugs, as replication of the Delta replicon lacking the S gene is potently inhibited by both drugs. This highlights the value of replicon systems that can recapitulate live-virus infection for the dissection of drug activity mechanisms.

## 4. Discussion

The Wuhan Hu-1 and Delta VOC dual-reporter replicons established in this study provide a useful tool for transient gene expression studies with a convenient read-out using two reporter proteins. Using smFISH the genome copy kinetics for one of the replicons was quantified and compared to a virus infection, confirming that replication of the replicon genomes was a suitable surrogate for virus replication. Additionally, this highlights genome replication of replicons is not the limiting step to establish stable cell lines. As the replicons lack the S gene, they provide a biosafe system to study the effects of drugs on intracellular virus replication. This was initially validated using the well-known pan-viral inhibitor remdesivir, comparing the Wuhan Hu-1 and Delta VOC replicons with ORF6-mScarlet and ORF7a-mNeonGreen replacements with rSARS-CoV-2 nsp1:KH-AA. Based on a luciferase reporter readout, the replicons successfully mirrored the sensitivity of the virus to remdesivir, with IC_50_ values obtained in the low micromolar range for both replicons and virus. These results are comparable to other reports [28,64].

Subsequently, this approach was used to investigate the mechanism of action of ritonavir and cobicistat, showing they are potent inhibitors of RepΔ-6mS replication. Interestingly, Rep-6mS did not show dose responsiveness to ritonavir and cobicistat. Further tests using the parental Delta VOC isolate used to establish RepΔ-6mS, a chimeric virus with the Wuhan S gene replaced with that of the Delta VOC (rSARS-CoV-2 S:Delta), rSARS-CoV-2 nsp1:KH-AA and rSARS-CoV-2 ORF6S, verified that the difference in phenotypes was not caused by (i) failure of the Delta VOC S to be inhibited by the drugs, (ii) inactivation of nsp1 translational shut-off by the K164A/H165A mutations or (iii) replacement of ORF6 with mScarlet. Taken together, the results imply that cobicistat and ritonavir appear to inhibit Delta VOC replication at multiple levels—inhibiting intracellular replication (as shown by the replicon data) and interfering with S-mediated entry (as shown with rSARS-CoV-2 S:Delta). In contrast, for Wuhan, their antiviral effect appears to be primarily S-dependent, with no clear inhibition of intracellular replication observed. Further investigation is required to determine if intracellular replication of the Delta VOC depends on either a viral protein or host factor specifically inhibited by both compounds. These findings highlight the utility of the SARS-CoV-2 replicons for drug screening and dissecting the mechanistic action of drugs. The study also demonstrates that the replicon system can be readily adapted to introduce VOC specific mutations and can replicate lineage-specific phenotypes.

A major aim of this study was to develop a cell line stably maintaining a SARS-CoV-2 replicon. Although the replicons produced in this study were replication competent, as shown by the expression of reporter genes replacing M, ORF6 or ORF7a, and by RNA copy kinetics, cells stably maintaining any of the replicons could not be isolated, despite repeated attempts using different cell lines and conditions. It has been reported that a SARS-CoV-2 replicon can be stably maintained in BHK-21 cells expressing the SARS-CoV-2 N protein. The nsp1 K164A/H165A mutations introduced into the replicon were found necessary to produce stable replicon cell clones [46]. Although these mutations were included in the replicons used in this study, cell populations stably maintaining the replicons could not be isolated, in line with the findings of another study [32]. Although the nsp1 K164A/H165A substitutions were stably maintained in the viral genome, they resulted in attenuated virus replication in a variety of cell lines, of both human and primate origin, as well as an increasing the sensitivity of the virus to the interferon response. Interestingly, rSARS-CoV-2 nsp1:KH-AA was still able to cause substantial CPE, albeit with a minor reduction in cytotoxicity compared to the wild-type virus at 24 hpi, with the effect becoming more pronounced by 48 hpi. However, as rSARS-CoV-2 nsp1:KH-AA has attenuated growth, the increased cell viability may be due to a slower progression of infection. This may partly explain why attempts to isolate a stable replicon-containing cell line were unsuccessful. The previously reported replicon cell line exhibited spontaneous mutations in nsp4 and nsp10 across nearly all replicon RNA populations isolated, which could suggest adaptive mutations were required for non-cytopathic replication in BHK-21 cells [46]. It would also be of interest to sequence the cellular genome, as the cells may harbour rare allelic variants that allowed for non-cytopathic or persistent replication of SARS-CoV-2 replicons.

Here, we provide a useful tool-kit of nsp1:KH-AA rSARS-CoV-2 replicons to assay viral RNA replication and provide characterisation of these systems in comparison to the parental recombinant virus, rSARS-CoV-2 nsp1:KH-AA. We validated these replicons as drug screening tools and demonstrated that comparable results to infectious virus can be obtained using these surrogate assays at CL2. Additionally, we highlight that these tools are useful to study drug activity mechanisms, particularly whether RNA replication or spread during infection may be inhibited by a compound.

## Data Availability

The data supporting the reported results are presented in Erdmann et al. Tables S1–S9 and Erdmann Supplementary Figures S1–S5.

## Supplementary Materials

The following supporting information can be downloaded at https://www.mdpi.com/article/10.3390/v17050597/s1, Erdmann_Supplementary Tables S1–S9.xls containing Table S1: SCoV-2 TAR fragments; Table S2: SCoV-2 Rep fragments; Table S3: OL-PCR primers; Table S4: Comp Wuhan v Delta; Table S5: inserted genes; Table S6: -rSARS-CoV-2; Table S7: Screening primers; Table S8: Electroporation; Table S9: Puromycin selection Erdmann Supplementary Figures S1–S5. Figure S1. Replicon positive cells identified after electroporation of mammalian cells with in vitro transcripts derived from Rep-Gp-RL and an N gene construct; Figure S2. Kinetics of replicon replication in VAT cells—raw luminescence values; Figure S3. Raw luminescence values corresponding to Figure 8 Panels A–D; Figure S4. Kinetics of the replication of in vitro RNA transcripts derived from the RepΔ-6mS replicon in Caco2A cells; Figure S5. Raw luminescence values corresponding to Figure 8 Panel E.

## Abbreviations

The following abbreviations are used in this manuscript:

CL3	Containment level 3
SARS-CoV-2	Severe acute respiratory syndrome coronavirus 2
CoV	Coronavirus
MERS	Middle Eastern respiratory syndrome
COVID-19	Coronavirus disease 19
UTR	Untranslated region
ORF	Open reading frame
sgRNA	Sub-genomic RNA
TRS	Transcriptional regulatory sequences
S	Spike
M	Membrane
E	Envelope
N	Nucleocapsid
HCoV-229E	Human CoV-229E
nsp1	Nonstructural protein 1
MHV	Murine hepatitis virus
ACE2	Angiotensin-converting enzyme 2
TMPRSS2	Transmembrane serine protease 2
VTN	VeroE6/TMPRSS2
VAT	VeroE6/ACE2/TMPRSS2
AAT	A549/ACE2/TMPRSS2
BHK-21	Baby hamster kidney 21
BAN	BHK/ACE2/N
BEAS-2BA	BEAS-2BA/ACE2
ALI	Air–liquid interface
TAR	Transformation-associated recombination
YSM-Trp	Yeast nitrogen base without amino acids supplemented with yeast synthetic drop-out medium minus tryptophan
nts	Nucleotides
eGFP	Enhanced green fluorescence protein
pac	Puromycin N-acetyl transferase
RLuc	Renilla luciferase
OL-PCR	Overlap-PCR
RT-PCR	Reverse-transcriptase PCR
VOC	Variant of concern
cDNA	Complementary DNA
E. coli	Escherichia coli
BAC	Bacterial artificial chromosome
BAC/YAC	Yeast artificial chromosome
LB	Lysogeny broth
PBS	Phosphate-buffered saline
hpt	Hours post-transfection
rSARS-CoV-2	Recombinant SARS-CoV-2
CPE	Cytopathic effect
MOI	Multiplicity of infection
MEM	Eagle’s minimal essential medium
MTT	Methylthiazolyldiphenyl-tetrazolium bromide
DPBS	Dulbecco’s PBS
smFISH	Single-molecule fluorescence in situ hybridisation
gRNA	Genomic RNA
DAPI	4′,6-diamidino-2-phenylindole
RLU	Relative luminescence units
dsRNA	Double-stranded RNA
hpi	Hours post-infection
SEM	Standard error of the mean
IFN	Type I interferon
IC_50_	Half maximal inhibitory concentration
JAK-STAT	Janus kinase-signal transducer and activator of transcription
DMSO	Dimethyl sulfoxide
