# Supplementary material for: A Novel Toolkit of SARS-CoV-2 Sub-Genomic Replicons for Efficient Antiviral Screening
Source: Viruses. 2025 Apr 23;17(5):597. doi: 10.3390/v17050597 (PMC12115450; doi:10.3390/v17050597)
Supplement: Supplementary file 1 [file viruses-17-00597-s001.zip › Erdmann_supplementary_v2/Erdmann Supplementary Figures.pdf]

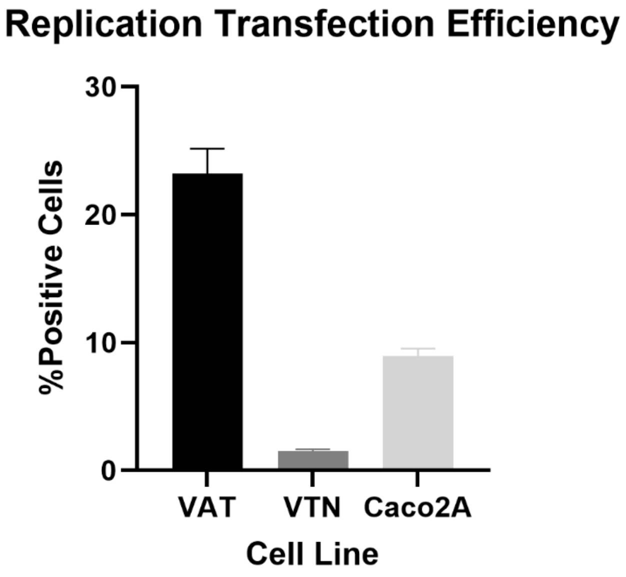

Figure S1. Replicon positive cells identified after electroporation of mammalian cells with in vitro transcripts derived from Rep-Gp-RL and an N gene construct. Mammalian cells were prepared for transfection using a Neon Transfection System using the settings outlined in Table S8. Cells were fixed and stained using an antibody recognising eGFP, and images scored for eGFP positive cells. The graphs show the mean and SD of n = 2 transfections.

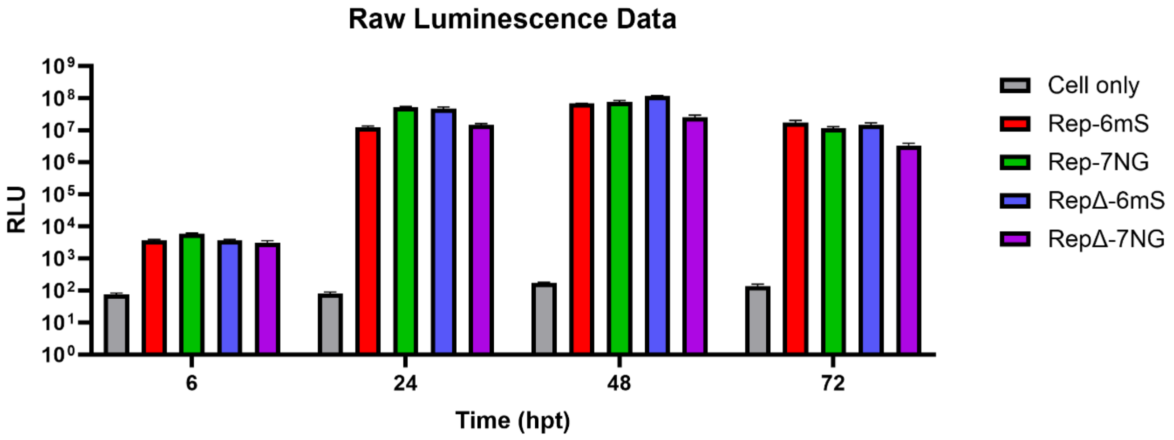

Figure S2. Kinetics of replicon replication in VAT cells - raw luminescence values. VAT cells were transfected with in vitro RNA transcripts corresponding to Rep-6mS, Rep-7NG, RepΔ-6mS and RepΔ-7NG and an N gene construct. At the indicated times post-transfection (hpt) the cells were assayed for RLuc activity in addition to a mock cell control. Graphs show the raw luminescence values with the mean and SEM of n=3 biological replicates.

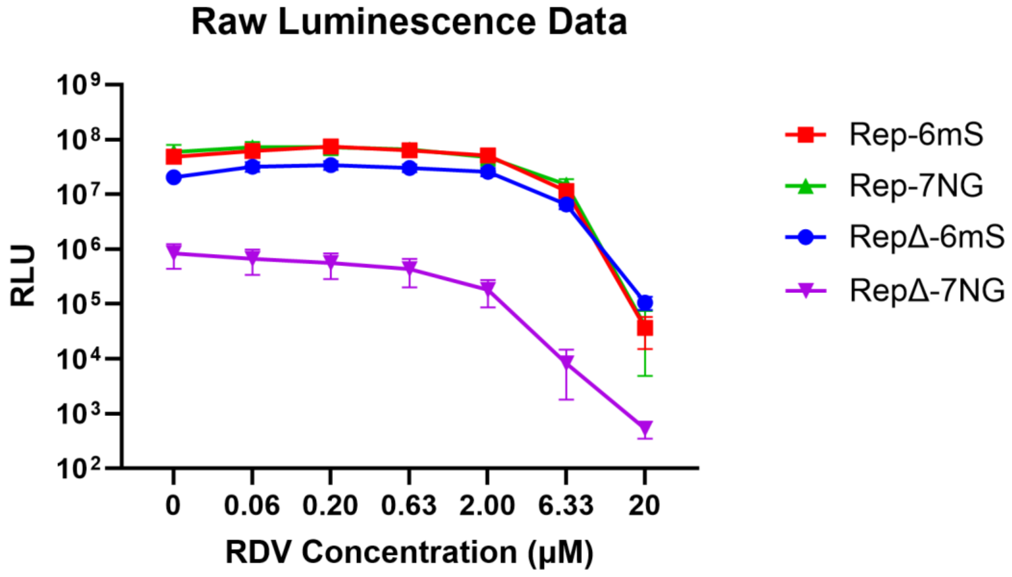

Figure S3 – Raw luminescence values corresponding to Figure 8 Panels A - D. Replicons are dose-responsive to remdesivir. Replicon dose-response curves to remdesivir. VAT cells were transfected with in vitro RNA transcripts corresponding to Rep-6mS, Rep-7NG, RepΔ-6mS and RepΔ-7NG and an N gene transcript. The transfected cells were incubated with a half-log fold dilution series of remdesivir (RDV), starting at 20 µM. At 24 hpt the cells were lysed and assayed for RLuc activity. The sample luminescence was adjusted for assay background by subtracting the cell only control. The graphs show mean and standard deviation of the RLU values obtained (n = 3-4).

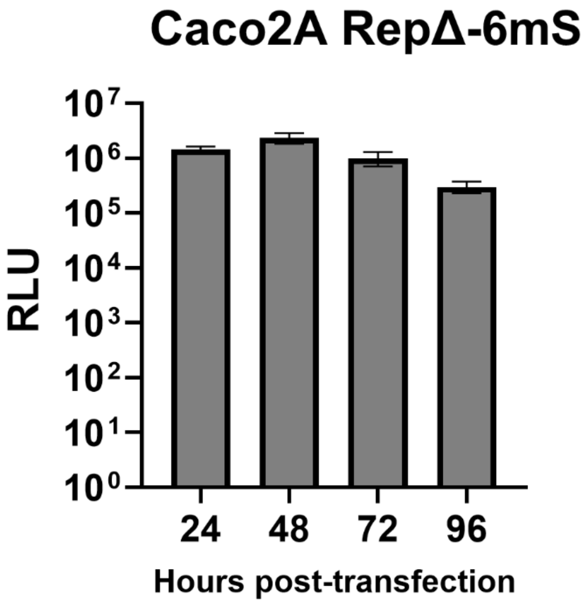

Figure S4. Kinetics of the replication of in vitro RNA transcripts derived from the RepΔ-6mS replicon in Caco2A cells. Caco2A cells were transfected with in vitro RNA transcripts corresponding to RepΔ-6mS and an N gene transcript. Expression of RLuc was monitored over the period 24-96 hours post-transfection. The sample luminescence was adjusted for assay background by subtracting the cell only control. Graphs show mean and SEM of n = 3 biological repeats.

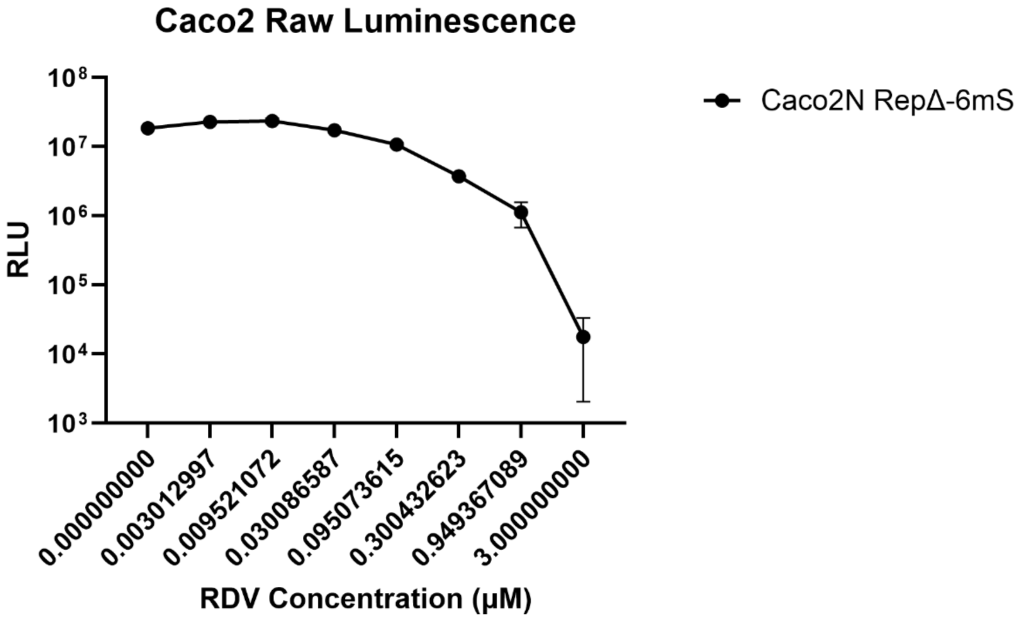

Figure S5. Raw luminescence values corresponding to Figure 8 Panel E. Caco2-N cells were transfected with in vitro RNA transcripts corresponding to RepΔ-6mS. The transfected cells were incubated with a half-log fold dilution series of remdesivir (RDV), starting at 3 μM. At 24 hpt the cells were lysed and assayed for RLuc activity. The sample luminescence was adjusted for assay background by subtracting the cell only control. The graphs show mean and standard deviation (n = 3) of the RLU values obtained.
